# Potential of *Pinus eldarica* Medw. tree bark for biomonitoring polycyclic aromatic hydrocarbons in ambient air

**DOI:** 10.1038/s41598-024-56182-3

**Published:** 2024-03-15

**Authors:** Sohrab Hasheminejad, Hossein Moradi, Mohsen Soleimani

**Affiliations:** Department of Natural Resources, Isfahan University of Technology, Isfahan, 8415683111 Iran

**Keywords:** Air pollution, Bioindicator, PAHs, PCA, Diagnostic ratio, Environmental sciences, Environmental chemistry, Ecology

## Abstract

Urban trees' biomonitoring of pollutants such as polycyclic aromatic hydrocarbons (PAHs) yields pertinent and useful data for air pollution management. The aim of this study was to biomonitor PAHs in pine (*Pinus eldarica* Medw.) trees in the city of Isfahan and identify their sources. In total, 34 samples of outer bark of the trees were collected and their contents of 16 EPA PAHs were analyzed. With a median value of 136.3 ng/g, the total PAH contents in tree barks varied from 53.4 to 705.2 ng/g. The average values of the diagnostic ratios for Ant/(Ant + Phe), Flu/(Flu + Py), BaA/(BaA + Chr) and IP/(IP + BP) were 0.19, 0.49, 0.45 and 0.49, respectively, revealing the PAHs majority source of pyrogenic. Meanwhile, principal component analysis showed two major types of PAHs sources including pyrogenic (fossil fuel combustion and industrial activities) and petrogenic (uncombusted) sources. The average ratio An/(An + Phe) and Flu/(Flu + Py) in bark samples was close to their relevant ratios in ambient air which demonstrated the potential use of this approach for biomonitoring of PAHs.

## Introduction

Air pollution is a worldwide concern since it has a negative impact on human health. Growing industrialization has exacerbated air pollution in recent years. Among the air pollutants, organic pollutants (e.g. polycyclic aromatic hydrocarbons (PAHs) have been more considered due to their properties such as toxicity, bioaccumulation, long-distance transport, and persistence. Increasing population growth, the use of fossil fuels, and the use of fertilizer or pesticides all contribute to an increase in organic pollutants in various environments^1^.

PAHs are an important class of organic pollutants that are emitted into the environment from a variety of sources, including pyrogenic, petrogenic, and biogenic sources. PAHs can be found in both gaseous and particulate forms. Because of their carcinogenic and mutagenic properties, they are classified as hazardous pollutants^2^. Because of potential health risks, the United States Environmental Protection Agency (EPA) designated 16 PAHs as priority pollutants (Table S1). For researchers, the proliferation of PAHs into the atmosphere is a human health concern. Monitoring and source identification of PAHs in the environment, on the other hand, is a critical issue for proper management by decision-makers and scientists. There are numerous monitoring strategies, but biomonitoring could be one of the best due to its economic efficiency and ease of use^3^. Biomonitoring is the process of taking samples of organisms or parts of organisms and providing relevant information on the quality of the environment^4^. Trees are employed as biomonitors to gather vital information such as the spatial or temporal distribution of pollution in a city^5–8^. The tree's components (stem, branches, leaves, and bark) can be easily utilized to monitor pollution levels in general^9^. Tree bark, on the other hand, is exposed to air pollution and accumulates pollutants in the outer layer of bark^10–13^. Tree bark is divided into two sections: inner bark and outer bark. The inner bark of older stems contains living tissue, but the outer bark contains dead tissue^14^. Since outer pine tree bark has a broad area of layered, porosity, and waxy dead cells, scientists consider it as a good accumulation biomonitor of PAHs to geographical distribution, source identification, and health evaluation^10,12,13,15–18^. Pollutants are absorbed by tree bark in a variety of methods, including air deposition, which is taken up by roots and leaves^9,19^. Because PAHs are somewhat soluble in water (Table S1) due to the high log K_ow_ index, uptake of PAHs by roots is unlikely^20^. *Pinus eldarica* Medw. is a common evergreen species in many cities such as Isfahan. Based on their qualities, pine trees are classified as tolerant in APTI, which indicates *P. eldarica* is not vulnerable to pollution^21^. The PAHs associated with particulate matters (PMs) are prevalent due to the bark structure of pine trees^22–24^. Diagnostic ratios (DRs) and principal component analysis (PCA) are common tools used in various studies^2,6,8,13,18,25–27^. The DRs technique is straightforward and effective for identifying pyrogenic or petrogenic sources. However, by integrating DRs data with PAH emission source ratios, it is possible to identify the relevant sources. PCA is a multivariate method that can be used to identify possible PAH sources. Understanding the markers of the various processes is essential in this method of source identification^18^.

Isfahan metropolitan is one the industrialized region in Iran having various environmental challenges such as water scarcity and air pollution together with their relevant pollutants and health risks. Soleimani et al. (2022) has found that PMs (particularly PM_2.5_) are a substantial pollutant in Isfahan city^28^. They found the concentration of 19 PAHs associated with PM_2.5_ in the range of 0.3–61.4 ng/m^3^. Furthermore, the highest and lowest PAHs concentrations were associated with summer and winter, respectively^28^. Accordingly, three sources (transportation, industrial activities, and natural gas combustion) were identified to be the main sources of PAHs associated with PM_2.5_ in Isfahan city. However, there are numerous activities from major industrial activities including power plants (which uses Mazot or natural gas), iron and steel plants (which uses coke and natural gas), oil refinery, petrochemical plant, brick manufacturers, as well as diesel and gasoline vehicles, which may be as source of PAHs emissions^28^.

In general, biomonitoring of the PAHs has some limitations such as high costs, methodological problems, and needs extensive samplings^4^. As a result, employing tree tissues as a biological indicator is one of the biomonitoring approaches that offers an advantage over the conventional methods. The main aims of this study were (i) to quantify the content of PAHs on outer *Pinus eldarica* Medw. (pine) bark, and (ii) to identify the potential sources of PAHs in Isfahan city using diagnostic ratios (DRs) and PCA methods, and (iii) comparison the result of source identification of the current study with those related to PAHs associated with ambient PM_2.5_. Providing this pertinent information might reveal the potential of outer pine barks as a reliable method for biomonitoring and PAHs source identification in ambient air which could be beneficial for superior air pollution management.

## Materials and methods

### Study area

Isfahan city is an industrialized city in central Iran (51° 39′ 40′′ E, 32° 38′30′′ N), with a proximate elevation of 1600 m and a land area of 480 km^2^. The climate is semi-arid, with maximum and lowest temperatures of 42 and − 12 degrees Celsius, respectively. The total amount of green space in Isfahan city is roughly 37 million m^2^, which is dispersed evenly throughout the city, and *P. eldarica* is a popular tree species in the city's green spaces^29^. In terms of air pollution sources, the city is surrounded by major industries such as cement and brick factories, iron and steel industries, power plants, oil refineries, and petrochemical facilities. The transportation system in Isfahan city uses gasoline and diesel on a regular basis^28^. Natural gas combustion is typically used as a source of energy for residential heating and cooking. Pollution is generally at its peak during the cold season due to inversion.

### Sampling design

**Figure 1:**
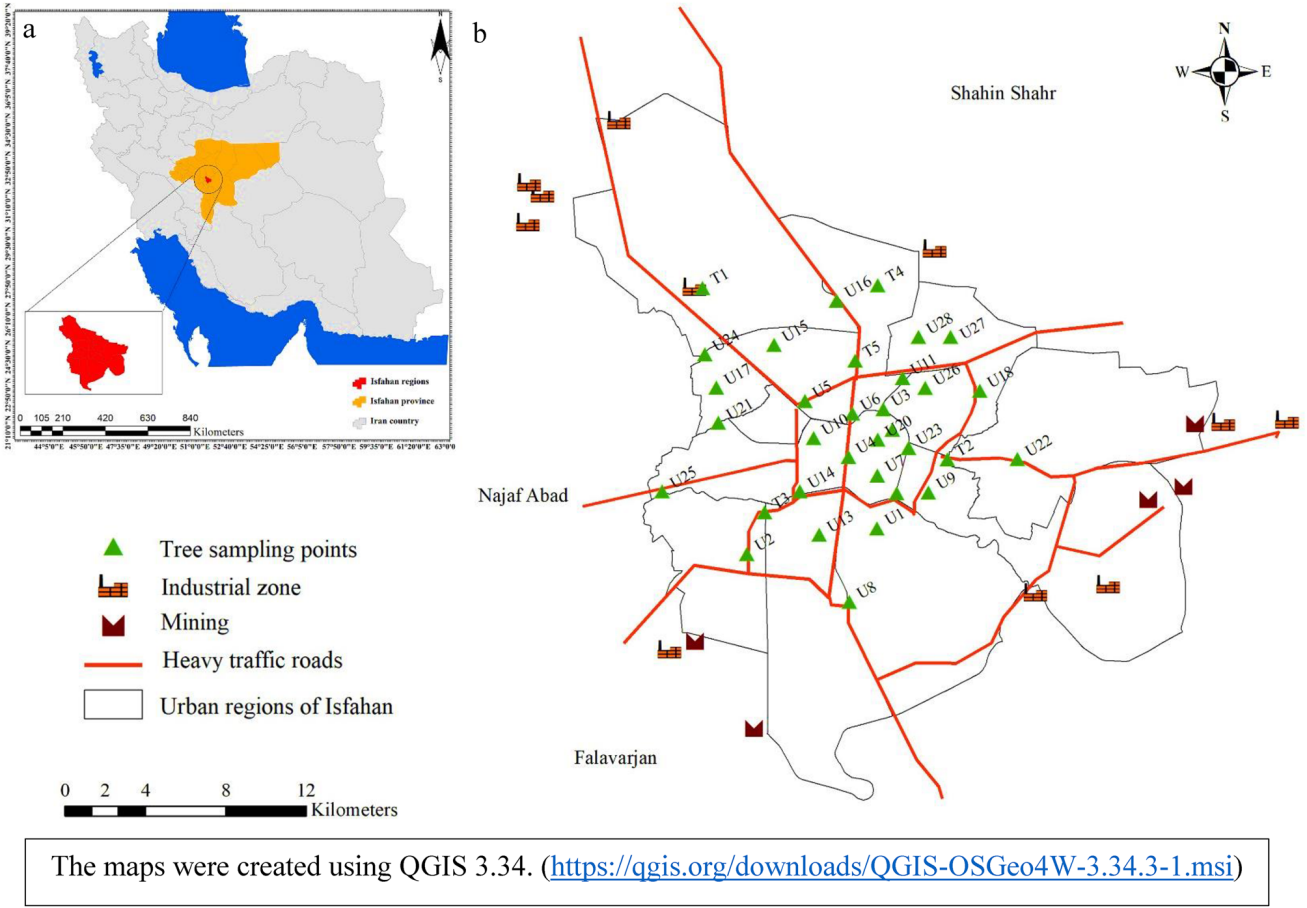
Study area. Location of Isfahan Province in the center of Iran (**a**). The sampling sites of pine tree barks distributed in Isfahan city (**b**).

In December 2020, 33 mature pine barks were collected in Isfahan city (Fig. 1). The *Pinus eldarica* Medw. tree, the synonym of *Pinus* *brutia* var. *eldarica* (Medw.) Silba, is a tall evergreen that can grow up to 12–15 m in height. It has an average growth rate of more than 1 m height per year and usually has six growth flushes and whorls annually. Additionally, its bole diameter at breast height increases at a rate of over 2 cm per year^30^. It features thick, brownish-gray bark that is layered. Although it is commonly used for its wood and paper as well as an important tree and an excellent choice for urban green spaces due to its ability to withstand tough climate and soil conditions^31^. However*, P. eldarica* is one of the most common trees worldwide which serves as a suitable bio-indicator in urban and industrial areas^32^. The researchers of the study with help of Mahnaz Bayat, a botanical expert, using voucher specimen number 11226, at the Herbarium of the Department of Natural Resources at Isfahan University of Technology, identified the tree species. A Voucher specimen from each sample has been deposited at the Herbarium under the number 11226. The choice was based on preliminary research and the findings of earlier studies on PM_2.5_ and associated with PM_2.5_ in Isfahan ambient air^28,33^. As a result, our judicial sampling strategy took into account population density, distance to industrial zones, and roadways. Bark samples with an area of 80 cm^2^ and a height of 1.5 m were taken from the street-side of the trees. All the bark samples were collected with official authorization from Isfahan Municipality under permission number 121.64825. After taking each sample, all bark samples were chopped with an adz and saw. The adz and saw were then cleaned with acetone to remove any organic impurities. The Anti-fungus was then used to fill the damaged area on the tree where the sample was taken. Finally, the samples were stored in polyethylene bags at a freezing temperature (about – 10 °C) and in the dark. An increment borer was used to collect core samples from the same trees. The age of the trees was calculated by counting the annual rings, which ranged from 14 to 57 years old. The ages and a brief description of the trees sampled are shown in Table S2.

### Preparation and extraction of samples

To prevent PAH loss, tree barks were freeze-dried in a freeze-dryer machine at – 40 °C for 24 h. A mixer device (Polymix PX-MFC90D) was used to finely grind the dry samples^34^. Following homogenization, the ground samples were stored in polyethylene bags in a dry, dark, and cool location^5^. Finally, 5 g of each sample was extracted for PAH analysis.

PAHs were extracted from bark samples using a sonication technique. In this method, 150 mL of acetone/dichloromethane 90:10 was extracted for 20 min, and 4 mL of PAH surrogate internal standard (Naphthalene-d8, Anthracene-d10, Chrysene-d12, Perylene-d12, and Acenaphthene-d10) mixture was added to all samples for QA/QC analyses (Table S3), and all sample volume was reduced to 2 mL by N_2_ stream^23,35^. All samples were cleaned using a silica gel column that had been deactivated with 5% deionized water. 40 mL dichloromethane was used to prewash the column. The extracted sample was then loaded into the column, and the PAHs were eluted with 40 mL dichloromethane. Finally, all samples were concentrated to 2 mL using a N_2_ stream and transferred to a GC vial^5^. The 16 EPA analyzed PAHs were naphthalene (Nap), acenaphthene (Acy), acenaphthylene (Ace), fluorene (Flu), phenanthrene (Phe), anthracene (Ant), fluoranthene (Fl), pyrene (Pyr), benzo(a)anthracene (B[a]A), chrycene (Chr), benzo(b)fluoranthene (B[b]F), benzo(k) fluoranthene (B[k]F), benzo(a)pyrene (B[a]P), indeno[1,2,3-cd]pyrene (I[c]P), dibenzo[a,h] anthracene (D[ah]A), and benzo [g,h,i]perylene (B[ghi]P).

### PAHs analyses

An Agilent 6890N gas chromatograph with a mass selective detector (Agilent 6890, inert MSD 5973) was used to test the samples for 16 EPA PAHs. The compounds were ionized by electron impact ionization. The capillary column was HP5-MS (30 m, 0.25 m, 0.25 mm), and the carrier gas was Helium at a flow rate of 1.2 mL/min. The data was collected using a specific ion monitoring approach. The limits of detection (LOD) are the lowest PAH concentrations that can be consistently detected. The lowest standard calibration curve and the area of a peak were used to compute LOD. Table S4 reveals PAH LODs, which ranged from 0.3 to 1.2 ng/g for all PAHs except naphthalene. Because of its high volatility and ease of evaporation, the LOD of this analyte was 2 ng/g.

### Source identification of PAHs

PAHs with 178 and 202 mass molecular weights are good for masking differences between combustion and petroleum sources, whereas PAHs with 228 and 276 mass molecular weights are less commonly utilized as PAH markers. Higher mass PAHs are usually derived from a variety of petroleum products such as crude oil, creosote, asphalt, and so on^35^. Therefore, to determine PAHs sources the ratio values of, 178 and 202 mass molecular PAHs such as An/(An + Phe), (anthracene to anthracene plus phenanthrene), Flu/(Flu + Pyr), (fluoranthene to fluoranthene plus pyrene), 228 and 276 mass molecular PAHs including BaA/(BaA + Chr), (benzo[a]anthracene to Benzo[a]anthracene plus chrysene), IP/(IP + BP), (Indeno[1,2,3-cd]pyrene to Indeno[1,2,3cd]pyrene plus Benzo[ghi]perylene), were used in different studies^27,36,37^. The standard values used to compare with estimated values in this investigation are shown in Table S3. Principal component analysis (PCA) is another method to identify PAHs sources. The PCA results demonstrate different component which show PAHs sources^8^. Package “factoextra” in R was implied for PCA.

### Ethical approval

The plant collection and use was in accordance with all the relevant guidelines.

## Results and discussion

### PAHs concentration in pine tree barks

**Figure 2:**
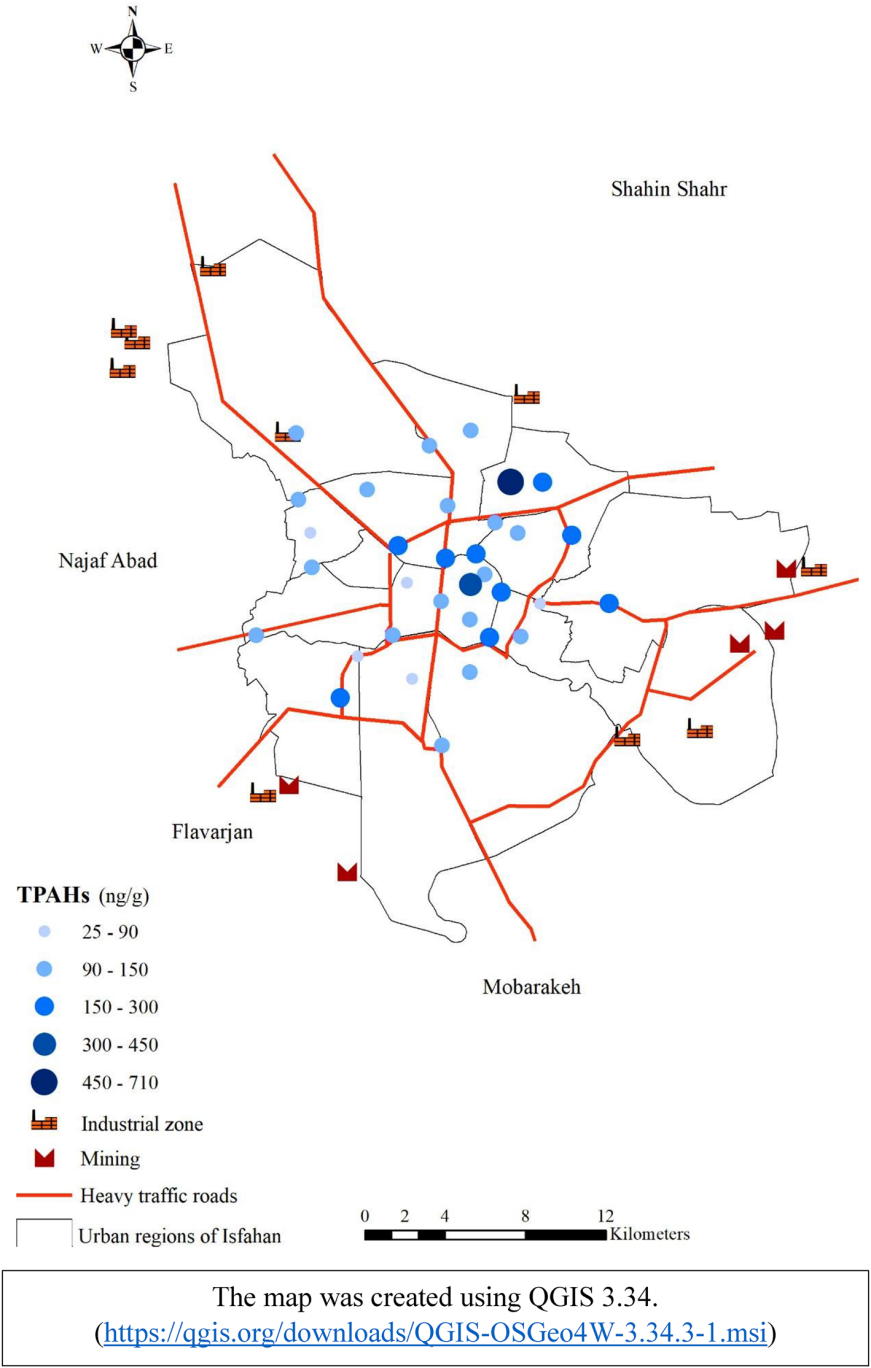
The spatial variation of ∑16 PAHs concentrations (ng/g) in pine tree barks of Isfahan city.

**Figure 3:**
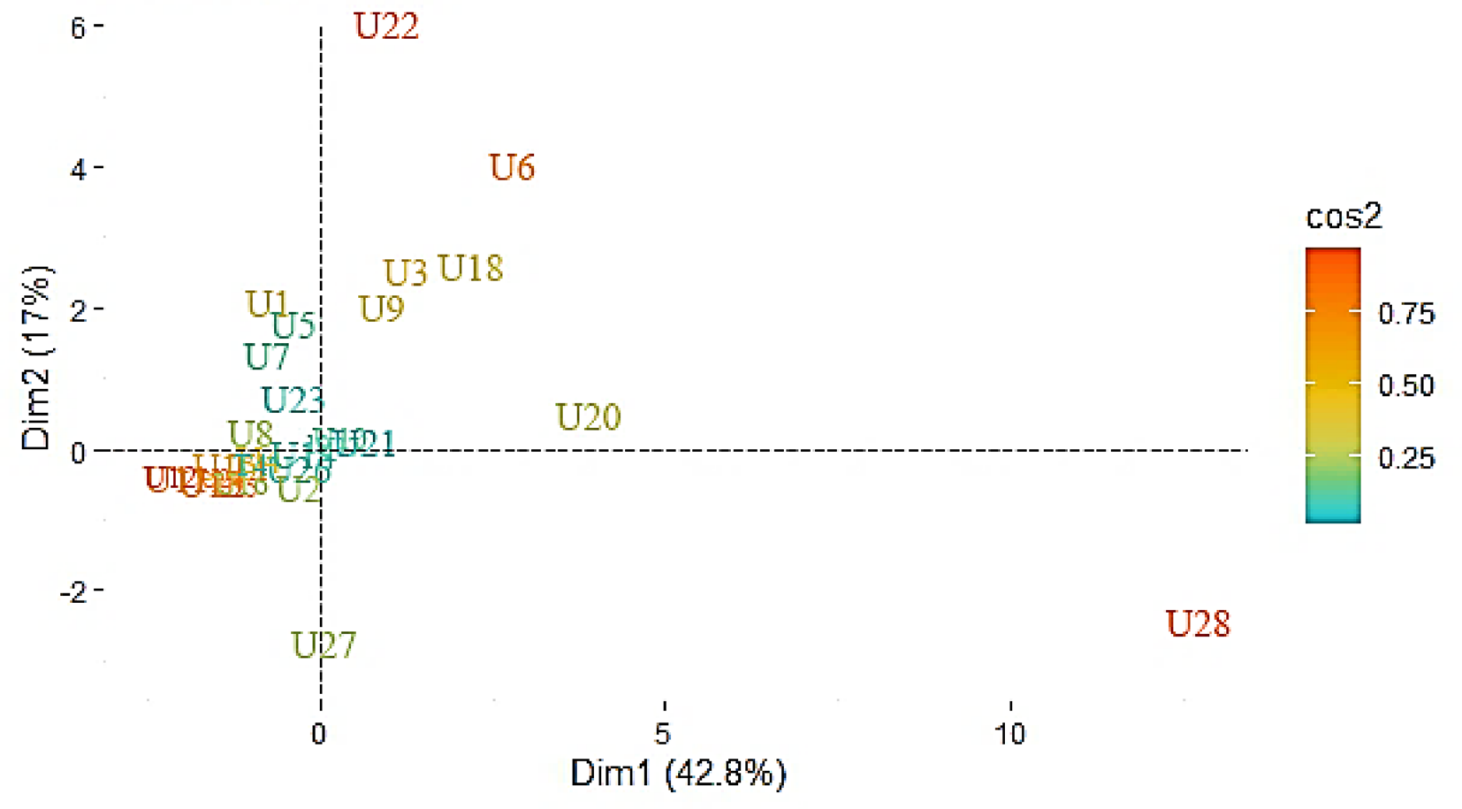
The PCA analysis of PAHs concentrations for sampling sites in pine tree barks in Isfahan city.

**Figure 4:**
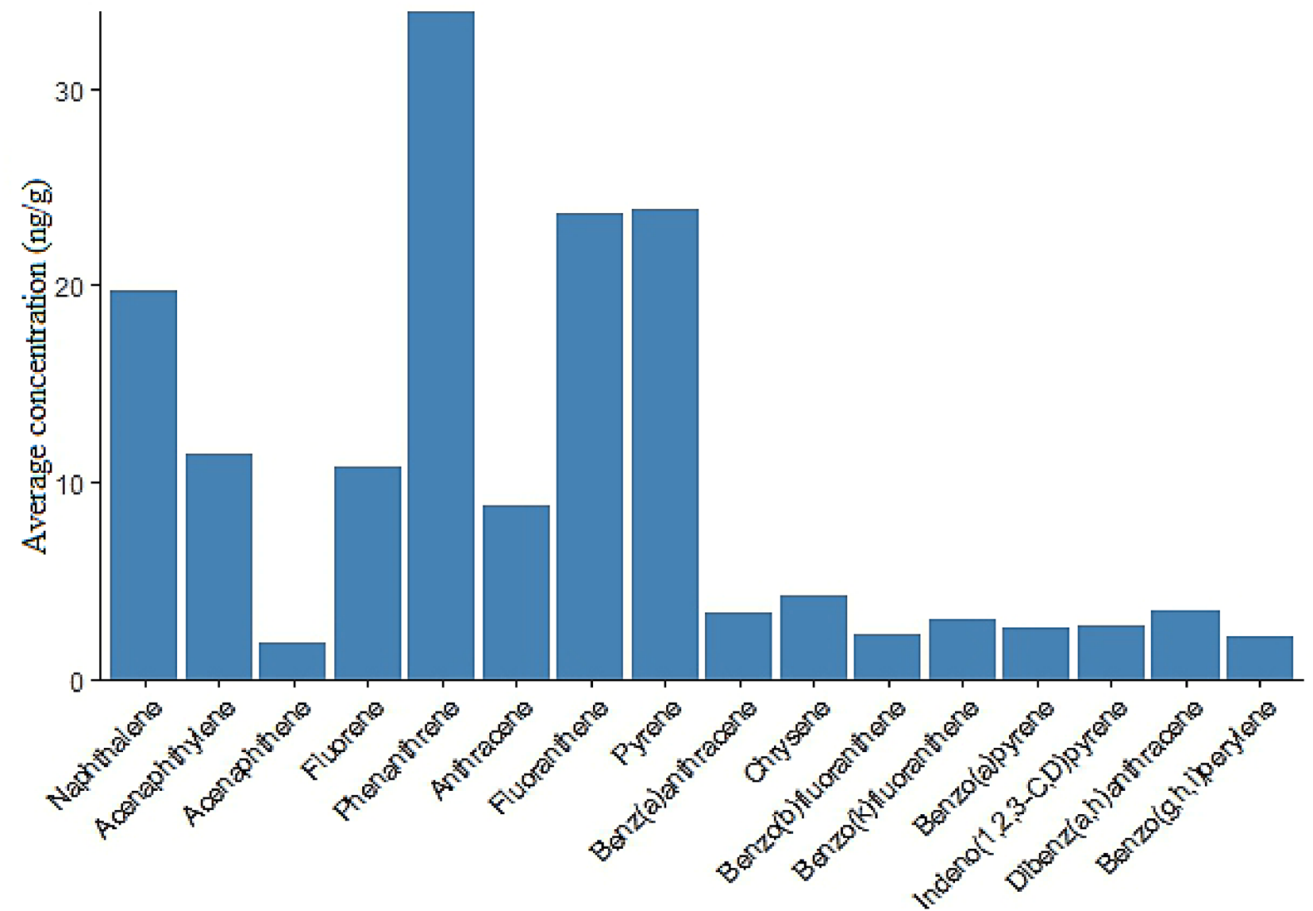
Average concentrations (ng/g) of individual PAHs in pine tree barks in Isfahan city.

The concentration of 16 PAHs in pine barks in Isfahan city ranged from 53.4 to 705.2 ng/g dw, with a mean value of 157 ng/g (Table 1). The highest PAHs concentration was found at site U28 (705.2 ng/g dw), which is influenced by a high traffic road as well as dozens of brick-making complexes. The second highest concentration was found in point U27 (290.7 ng/g dw), which is near a road with high traffic volume (Fig. 2). The results of PCA analysis showed the U22 sampling site is located beside a highway and is separated from other sampling sites. Similarly, U6 and U20 sampling sites located in the city center showed a similar pattern (Fig. 3). U6 is located in a residential area and it may also be influenced by heavy traffic during the day. U22 is located in a crowded square nearby a traditional Bazaar as well as a bus terminal. Therefore, these results revealed that the sources of the PAHs in the city might vary depending on the location. Soleimani et al. (2022) found that the sources of PAHs in this metropolis do not change much over seasons because they are mostly sourced by transportation, industrial activities and combustion of fossil fuels^28^. However, during the cold season, PAH concentrations may change due to climate conditions such as temperature inversion. In addition to the source type, other factors such as distance, mereological parameters, and photolytic or biological degradation can influence the PAH concentration^38^. The atmosphere of an urban area may be confined by buildings, trees, and other impediments, allowing PAHs to be deposited on various surfaces such as barks. A comparison between our findings with others presented in Table 2. The PAHs concentrations were stated higher in those studies than in the current study. Based on the average concentrations of individual PAHs, we found that naphthalene, acenaphthylene, fluorene, phenanthrene, and anthracene, which are 2–3 rings components, are predominate (Fig. 4). Then, the four-rings components including fluoranthene and pyrene take the lead. Other individual PAHs had an average concentration of less than 10 ng/g, which is comparable to the previous studies^5,18,34^. Several studies revealed that the concentration of low molecular weight PAHs measured in various plant tissues might be higher than the high molecular weight PAHs, due to the varied K_ow_ of the individual PAHs which affect PAHs sorption by plants^5,9,12,18^.

**Table 1:** The statistical summary of PAHs in pine tree barks in Isfahan city (ng/g dw).

PAHs	Mean	Std deviation	Total	Minimum	Q1	Median	Q3	Maximum	Range
Nap	19.77	39.57	652.28	1.22	5.52	10.73	16.91	223	221.78
Acy	11.39	8.21	375.98	0.3	5.89	8.24	15.7	34.84	34.54
Ace	1.887	1.32	62.281	0.3	1.21	1.454	1.815	5.806	5.506
Fl	10.76	7.84	355.2	4.41	6.42	9.14	12.29	48	43.59
Phe	33.9	24.79	1118.6	7.2	17.79	30.43	38.21	125	117.8
Ant	8.79	12.44	290.13	1.06	2.33	3.58	8.41	60	58.94
Flu	23.63	20.18	779.81	3.77	12.71	16.82	25.11	89.36	85.59
Py	23.81	22.11	785.71	2.76	12.09	16.72	27.59	98	95.24
BaA	3.416	2.589	112.719	0.2	1.697	3.144	4.427	13	12.8
Chr	4.24	3.767	139.914	0.4	1.832	3.202	4.952	14	13.6
BbF	2.281	2.088	75.261	0.4	0.572	1.852	3.079	10	9.6
BkF	3.05	8.5	100.49	0.4	0.58	1.36	2.36	50	49.6
BaP	2.596	2.46	85.66	0.6	0.6	2.143	3.627	13	12.4
IP	2.743	3.257	90.519	1.2	1.2	1.2	2.459	15.805	14.605
DaA	3.48	6.61	114.98	1.2	1.2	1.2	1.2	34.24	34.24
BP	2.192	2.216	72.345	1.2	1.2	1.2	1.2	9.04	7.84

**Table 2:** Comparison of ∑PAHs in tree bark of different geographical regions to the results of the current study (ng/g dw).

Site, country	Numbers of congeners	PAHs concentration (ng/g)	References
Jiangsu province, China	15	1560–6.18	Zhou et al. (2014)
Yangtze plain, China	15	1300–27	Wu et al. (2019)
Rural area, China	16	3803–6.3	Niu et al. (2019)
Sicily, Italy	19	1015–33	Orecchio et al. (2008)
Bursa, Turkey	14	593–81	Sari et al. (2021)
Pohang, South Korea	16	124–26	Choi et al. (2014)
Sao Paulo, Brazil	16	1640–242	Pereirra et al. (2007)
Current study	16	705.2–53.4

### Sources of PAHs in pine tree bark

**Figure 5:**
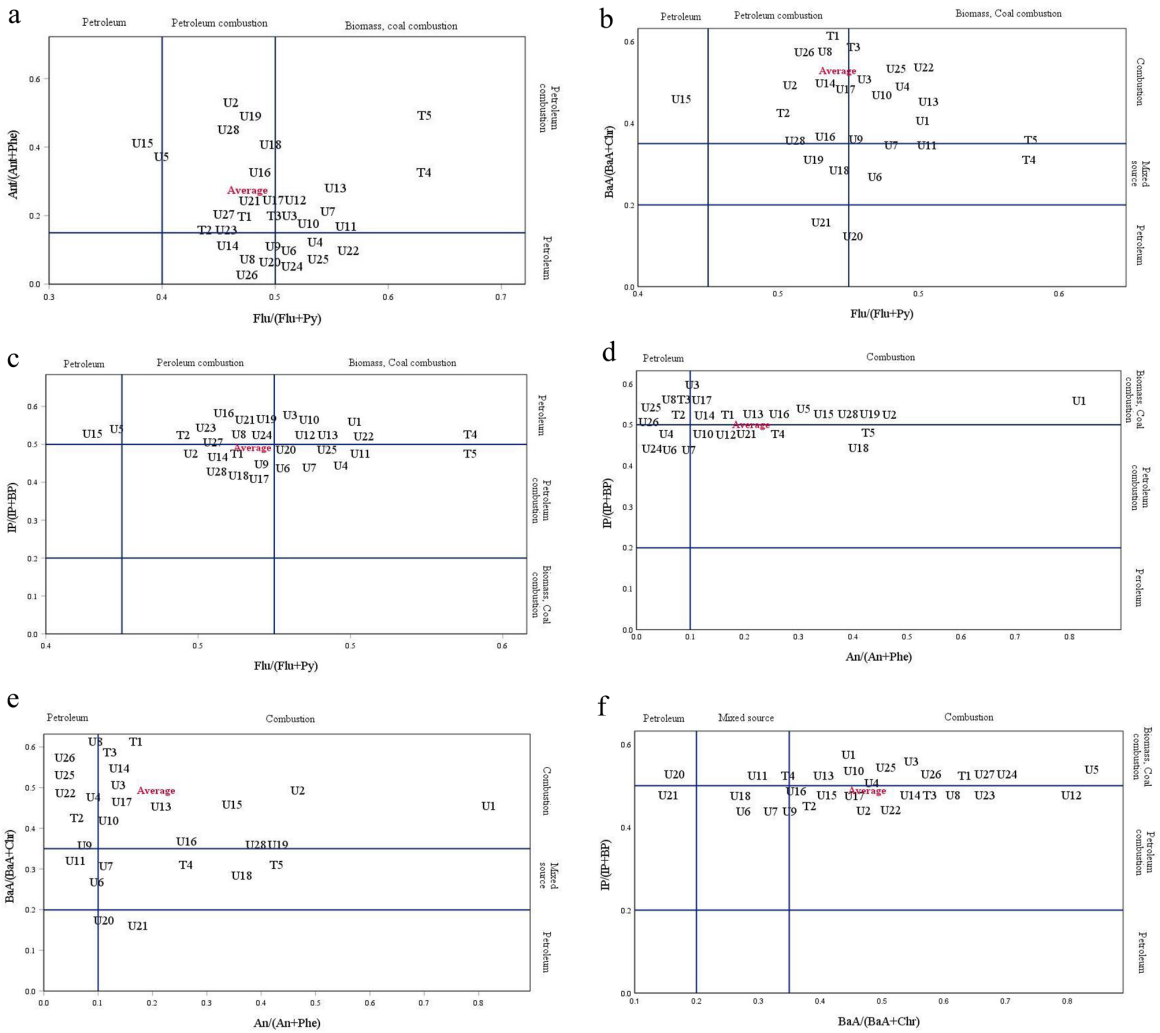
Cross plots of diagnostic ratios (DRs) of PAHs in pine tree barks in Isfahan city.

Cross plots of diagnostic ratios (DRs) of PAHs in pine barks from Isfahan city presented in Fig. 5. The threshold of 0.1 is as a reference value of the An/(An + Phe) ratio. Based on the threshold, values less and more than 0.1 indicate petrogenic and pyrogenic sources, respectively^36^. Our results showed the ratios were ranged from 0.06 to 0.79, with mean values of 0.19. It demonstrates that the majority of the sites in Isfahan city are exposed by pyrogenic sources. Flu/(Flu + Py) is another ratio used for diagnosis. In relation to this ratio, petroleum is represented by values < 0.4, liquid fossil fuel combustion is represented by values < 0.5, and biomass or coal combustion is indicated by values > 0.5^34^. The calculated ratio of PAHs in pine bark has a mean value of 0.49 and a range of 0.37–0.62. This ratio illustrates the role of vehicles powered by liquid fossil fuel. According the ratio (BaA/BaA + Chr), the values < 0.2 indicate the petrogenic sources, 0.2 < values < 0.35 indicate the mixed sources of petrogenic and pyrogenic, and values > 0.35 indicate the combustion sources^34^. According to our findings, the ratio ranged from 0.13 to 0.82, with an average of 0.45, suggesting that one of the main sources of PAHs in the city of Isfahan is pyrogenic. The fact that additional studies found that the values for this ratio can range from 0.22 to 0.55 for gasoline combustion and 0.38–0.64 for diesel combustion further highlights the significance of fossil fuels combustion in Isfahan city^39^. Values less than 0.2 indicate petrogenic sources, values between 0.2 and 0.5 indicate liquid fossil fuel combustion, and values more than 0.5 indicate grass, wood, or coal combustion^36^. The estimated ratio of this study ranged from 0.46 to 0.53, with a mean value of 0.49. This ratio also indicates that pyrogenic sources are the most prevalent (Table S5). The pattern of PAH dispersion may be influenced by a number of factors, including temperature and photolysis, therefore the results of DRs may not pinpoint precise sources.

In Table 3, a comparison of the diagnostic ratios of PAHs in the pine bark and those measured in PM_2.5_ samples from the same region^28^ is presented. The ratios of 178 and 202 mass molecular PAHs in pine tree bark were nearly identical to earlier ratios of PM_2.5_ samples. In bark samples, the average An/(An + Phe) and Flu/(Flu + Py) were 0.19 and 0.49, respectively. However, the mean values of these ratios in ambient PM_2.5_ samples was 0.18 and 0.43, respectively, indicating that the results of these investigations likely demonstrated that pyrogenic sources are predominated across the city. As a result, employing *Pinus eldarica* Medw barks for PAHs biomonitoring using the mentioned diagnostic ratios may have a high potential to identify PAHs sources.

**Table 3:** The comparison of diagnostic ratios (DRs) in tree bark samples and ambient PM_2.5_ in Isfahan city^28^.

Ratios	PAHs in PM_2.5_		PAHs pine bark	
	Mean	Min–Max	Mean	Min–Max
An/(An + Phe)	0.18	0.34–0.09	0.19	0.79–0.06
Flu/(Flu + Py)	0.43	0.58–0.32	0.49	0.62–0.37
BaA/(BaA + Chr)	0.34	0.43–0.17	0.45	0.82–0.13
IP/(IP + BP)	0.31	0.41–0.22	0.49	0.53–0.46

**Figure 6:**
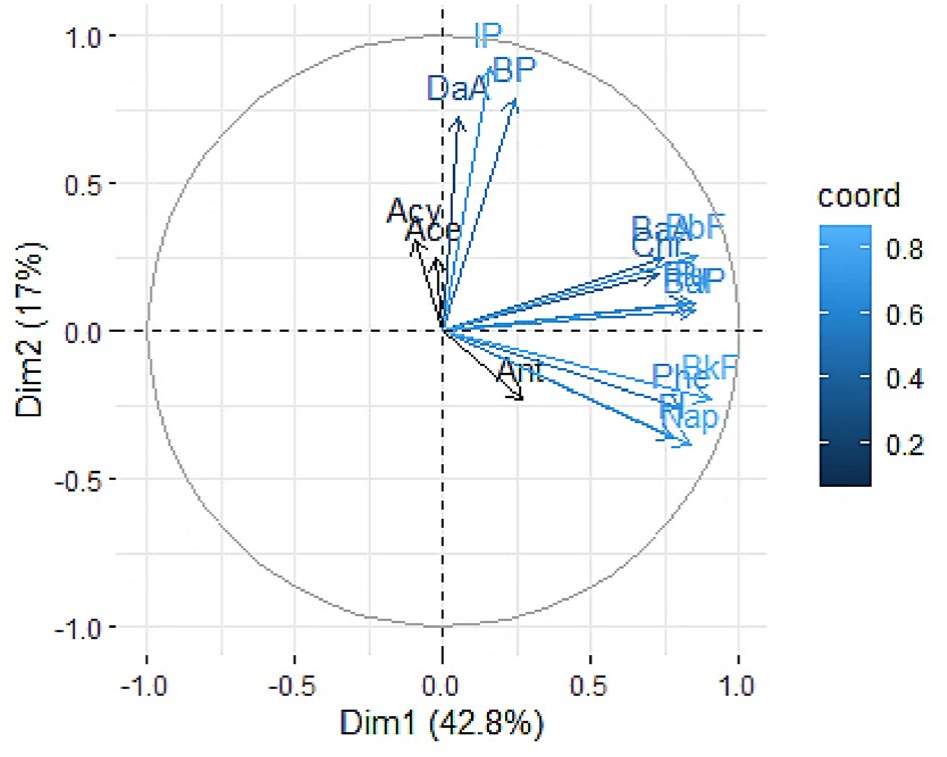
PCA analysis for PAHs concentrations in pine tree barks in Isfahan city.

The PCA results showed four components of relevant PAHs in pine barks of Isfahan city (Fig. 6), where the first, second, third, and fourth components were explaining 42.7%, 17.08%, 10.83%, and 9.4% of total variance, respectively (Table S6). The first component is mostly loaded by PAHs such as BkF, BbF, Py, BaP, Nap, Flu, Phe, Fl, BaA, and Chr, which are mainly markers of industrial activities, diesel, and natural gas combustion^37,40^. Moreover, Fluorene, Py, Chr, BaA, BeP, and BaP were identified as markers of industrial activities^35,37^. Around Isfahan city, there are several major industrial zones with a high capacity of producing PAHs, such as power plants, iron and steel plants, brick and cement factories^28,40^. The first component contains Phe, Fl, BaA, Chr, and Py which are known as indicators of natural gas combustion^35^. Natural gas is not only utilized for residential heating and cooking in Isfahan city, but it is also used as a fuel in major factories such as power plants and steel plants. Another source of PAHs is diesel combustion, and the markers are BkF, BbF, and BaP^35,37^. In Isfahan city, diesel fuel is widely used in public transportation and trucks as well as industrial plants^28^.

The second component related to the combustion of gasoline. indeno[1,2,3-cd]pyrene (I[c]P), BP, and DaA were identified as gasoline combustion markers ^35,41^. Three PAHs were considered as petrogenic markers such as Acy, Ace, and Ant which are found in the third and fourth components ^42^. Overall, the findings suggested that the main sources of PAHs in Isfahan City were likely natural gas, as well as diesel and gasoline combustions.

According to the PAHs sources identification, Isfahan is mostly affected fuel combustion in various sources including transportation and industrial activities and their related transportation to move the raw materials, products, and the personnel.as well as power plants and residential heating systems. The Isfahan city witnesses more than a million private gasoline cars and 2500 public diesel vehicles passing through it every day^28^. Being a highly industrialized city in Iran, Isfahan is surrounded by major industries, including brick factories, iron and steel plants, power plants, oil refineries, and petrochemical facilities. Therefore, industrial activities and transportation as two anthropogenic sources could be the most significant sources of PAHs in ambient air^28^.

PC1 values were high in both U28 and U20 (Fig. 3). U28 is near brick manufacturing zones and the roads with heavy traffic. The point U20 is located in a crowded square surrounded by commercial activity, and car traffic which has expanded dramatically during the last decade. This point may be significantly influenced by the pyrogenic sources such as natural gas, gasoline and diesel combustion. Points U5, U1, and U22 all exhibited high PC2 values, indicating significant gasoline combustion at these sites. U5 and U22 were assigned to the highway side, whilst U1 was assigned to the street side. Samples from the three green spaces (U3, U9, and U18) had high PC1 or PC2 scores. Green spaces have an important role in capturing PAHs^43^. Ant, Phe, Flu, and Py concentrations of the tree barks were higher at these sampling sites than those from the others. Thus, the trees could sorb PAHs from the environment, demonstrating their potential in purifying the urban air from those compounds.

## Conclusion

The concentration of 16 PAHs in pine barks in Isfahan city ranged from 53.4 to 705.2 ng/g dw, with a mean value of 157 ng/g, where three and four-ring PAHs were predominated at all sites. The source identification of PAHs in pine barks and recent research yielded nearly identical results, indicating that vehicle emissions, natural gas combustion, and industrial activities were the major PAHs sources in Isfahan city during the cold season. The diagnostic ratios of PAHs from tree barks and ambient PM_2.5_ in Isfahan city (particularly for An/(An + Phe) and Flu/(Flu + Py) ratios) showed the same possible sources of the compounds. Biomonitoring of PAHs using bark of *Pinus eldarica* Medw. could be a cost-effective, more reasonable, and applicable approach than conventional monitoring. The diagnostic ratios and PCA analysis revealed the pyrogenic source of PAHs in the pine tree barks. Although pyrogenic sources are the main source of PAHs emissions in the city, petrogenic sources (i.e. non-combustion sources) should be considered in air pollution management.

### Supplementary Information

Supplementary Tables.

## Data Availability

The datasets generated analyzed during the current study are available from the corresponding author on reasonable request.
