# Supplementary material for: Potential of Pinus eldarica Medw. tree bark for biomonitoring polycyclic aromatic hydrocarbons in ambient air
Source: Sci Rep. 2024 Mar 15;14:6259. doi: 10.1038/s41598-024-56182-3 (PMC10943078; doi:10.1038/s41598-024-56182-3)
Supplement: Supplementary file 1 — Supplementary Tables. [file 41598_2024_56182_MOESM1_ESM.docx]

| Table S1. molecular weight, log K_ow_ and water solubility of 16 EPA PAHs [5] | | | |
| --- | --- | --- | --- |
| Log K_ow_  C˚25 | Water solubility (mg/L) C˚25 | Molecular weight  (g) | component |
| 3.37 | 31 | 128.17 | Nap |
| 4 | 16 | 152.19 | Acy |
| 3.92 | 3.8 | 154.21 | Ace |
| 4.18 | 1.9 | 166.22 | Fl |
| 4.46 | 1.1 | 178.23 | Phe |
| 4.49 | 0.04 | 178.23 | Ant |
| 8.9 | 0.2 | 202.25 | Flu |
| 8.8 | 0.13 | 202.25 | Py |
| 5.8 | 0.011 | 228.29 | BaA |
| 5.73 | 0.0019 | 228.29 | Chr |
| 5.78 | 0.0015 | 252.31 | BbF |
| 6.5 | 0.0008 | 252.31 | BkF |
| 6.35 | 0.0015 | 252.31 | BaP |
| 6.5 | 0.0005 | 278.35 | DaA |
| 6.63 | 0.00014 | 276.33 | BP |
| 6.7 | 0.00019 | 276.33 | IP |

| Table S2. Description of sampling sites and age of trees in Isfahan city. | | |
| --- | --- | --- |
| Sampling site | The age of tree | Description |
| U1 | 57 | A relatively low-traffic street, the sample was taken at a distance of 3 meters from the street. |
| U2 | 26 | There is an empty space around this area with no residential area nearby. The flower and plant market used to be located in the south of this park. |
| U3 | 37 | Green space on the edge of Quds intersection |
| U4 | 40 | High traffic density in this area during the day is due to the proximity of the municipal building, which is located 6 meters away from the main street. |
| U5 | 14 | The tree is located 2 meters away from the highway on both sides. |
| T1 | 21 | Sampling was conducted in the green area located north of the terminal and south of Amir-Kabir Street. |
| U6 | 35 | Residential area could be seen at the edge of the square and the entrance of Kaveh Street, east of the tree. |
| U7 | 24 | At the first intersection of the entrance to Sharif Waqfi Street, the distance from the street is 2 meters. |
| U8 | 38 | There is at the entrance of the residential complex. |
| T2 | 20 | in front of the entrance of the terminal |
| U9 | 26 | Located within the Ghadir green space complex, this area experiences high traffic due to the presence of various offices. |
| U10 | 34 | On the edge of the intersection, half a meter away from the street |
| U11 | 29 | In the northern part of the highway, there were small industrial workshops and residential areas. |
| U12 | 25 | On the side of the street, there was a crowd and traffic because of the commercial centers. |
| U13 | 32 | On the edge of the intersection, the presence of residential areas and small workshops around |
| U14 | 45 | This street has heavy traffic due to workshops and mechanical guilds. |
| U15 | 32 | There are residential areas and gas stations around this park, and it is one of the most populated places in Isfahan. |
| U16 | 17 | There was a residential area around it and a low-traffic street in the southern part of the tree. |
| T3 | 28 | The sample was taken inside the terminal. |
| T4 | 27 | In addition to buses, heavy vehicles can also be seen at this terminal at night. Additionally, there is a tree located in the southern part of Farzangan highway. |
| U17 | 30 | There is plenty of open space as the land around it is used for agriculture. |
| U18 | 17 | There is a green space located 300 meters east of a residential area and Shahid Agha Babaei highway. |
| T5 | 23 | The location referred to is situated in the northern part of the terminal and to the west of Kaveh highway. |
| U19 | 37 | The tree is located on the edge of the plot and 20 meters away from the taxi station. |
| U20 | 35 | In the last two decades, there has been an increase in car traffic due to the development of the field. |
| U21 | 40 | Apart from vehicle traffic, the street houses small production centers, foundries, and agricultural lands. |
| U22 | 36 | The residential area around the tree is located on the side of highway and above the sugar factory. |
| U23 | 43 | During office hours, the traffic of vehicles increases on the outskirts of the square, which is home to commercial and electronic centers. |
| U24 | 25 | 300 meters from Imam Khomeini highway in the green area |
| U25 | 28 | On the side of the highway and in front of the Najvan Forest Park |
| U26 | 14 | Near Sheikh Toosi intersection, in front of Parvin Etesami's green space |
| U27 | 25 | There are two parking lots near the Zainabiyah shrine at the intersection, close to the brick industry. They are located about 1 meter away from the main streets on each side. |
| U28 | 38 | In the open area with multiple buildings, there are casting workshops located 2 meters away from the main street. This site is near bricks factory |

| Table S3. List of internal and for the quantification of PAHs. | |
| --- | --- |
| Internal standard | PAH |
| Naphthalene-d_8_ | Naphthalene |
| Acenaphthene-d_10_ | Acenaphthene, Acenaphthylene, Fluorene |
| Phenanthrene-d_10_ | Anthracene, Fluoranthene, Phenanthrene |
| Chrysene-d_12_ | Benzo(a)anthracene, Chrysene, Pyrene |
| Perylene-d_12_ | Benzo(b)fluoranthene, Benzo(k)fluoranthene, Benzo(g,h,i)perylene, Benzo(a)pyrene, Dibenz(a,h)anthracene, Indeno(1,2,3-cd) pyrene |

| Table S4. Limit of detection (LOD) of PAHs (µg/kg) in pine barks | |
| --- | --- |
| PAHs | LOD |
| Naphthalene | 2.0 |
| Acenaphthylene | 0.30 |
| Acenaphthene | 0.30 |
| Fluorene | 0.30 |
| Anthracene | 0.60 |
| Phenanthrene | 0.30 |
| Pyrene | 0.30 |
| Fluoranthene | 0.30 |
| Chrysene | 0.20 |
| Benzo(a)anthracene | 0.40 |
| Benzo(k)fluoranthene | 0.40 |
| Benzo(b)fluoranthene | 0.40 |
| Benzo(a)pyrene | 0.60 |
| Dibenzo(a,h)anthracene | 1.20 |
| Benzo(g,h,i)perylene | 1.20 |
| Indeno(1,2,3-c,d)pyrene | 1.20 |

| Table S5**.** Selected molecular ratios of PAHs used for source identification. | | | |
| --- | --- | --- | --- |
| Reference | source | threshold | Ratio |
| Yunker et al. (2002) | Petrogenic | <0.1 | Ant/(Ant+Phe) |
|  | Pyrogenic | >0.1 |  |
| Yunker et al. (2002) | Petrogenic | <0.4 | Flu/(Flu+Py) |
|  | Fossil fuel combustion | 0.4 – 0.5 |  |
|  | Grass, Wood, Coal Combustion | >0.5 |  |
| Yunker et al. (2002) | Petrogenic | <0.2 | BaA/(BaA+Chr) |
|  | Mixed source of Petrogenic and Pyrogenic | 0.2 – 0.35 |  |
|  | Pyrogenic | >0.35 |  |
| Yunker et al. (2002) | Petrogenic | <0.2 | IP/(IP+BP) |
|  | Liquid fuel combustion | 0.2 – 0.5 |  |
|  | Grass, Wood and Coal Combustion | >0.5 |  |

| Table S6. The relation between PAHs in PCA results in *Pinus eldarica* in Isfahan city | | | | | |
| --- | --- | --- | --- | --- | --- |
| PAHs |  | Component | | | |
|  |  | 1 | 2 | 3 | 4 |
|  | Percent of Variance | 42.701 | 17.085 | 10.863 | 9.403 |
| BkF | | 0.900 |  |  |  |
| BbF | | 0.857 |  |  |  |
| Py | | 0.855 |  | 0.364 |  |
| BaP | | 0.841 |  |  |  |
| Nap | | 0.831 | -0.386 | -0.304 |  |
| Fl | | 0.819 |  | 0.417 |  |
| Phe | | 0.802 |  |  |  |
| Flu | | 0.773 | -0.366 |  |  |
| BaA | | 0.740 |  |  | -0.375 |
| Chr | | 0.724 |  |  |  |
| IP | |  | 0.907 |  |  |
| BP | |  | 0.770 |  |  |
| DaA | |  | 0.739 | -0.458 |  |
| Acy | |  |  | 0.743 | 0.413 |
| Ace | |  |  | 0.501 | -0.309 |
| Ant | |  |  |  | 0.858 |
